# The unique fold and lability of the [2Fe-2S] clusters of NEET proteins mediate their key functions in health and disease

**DOI:** 10.1007/s00775-018-1538-8

**Published:** 2018-02-12

**Authors:** Ola Karmi, Henri-Baptiste Marjault, Luca Pesce, Paolo Carloni, Jose’ N. Onuchic, Patricia A. Jennings, Ron Mittler, Rachel Nechushtai

**Affiliations:** 1 The Alexander Silberman Life Science Institute and the Wolfson Center for Applied Structural Biology, The Hebrew University of Jerusalem, Edmond J. Safra Campus at Givat Ram, 91904-0375 Jerusalem, Israel; 2 Computational Biomedicine Section, Institute of Advanced Simulation (IAS-5) and Institute of Neuroscience and Medicine (INM-9), Forschungszentrum Jülich GmbH, 52425 Jülich, Germany; 3 Department of Physics, RWTH-Aachen University, 52056 Aachen, Germany; 4 Center for Theoretical Biological Physics, Rice University, Houston, TX 77005 USA; 5 Departments of Physics and Astronomy, Chemistry and Biosciences, Rice University, Houston, TX 77005 USA; 6 Department of Chemistry and Biochemistry, University of California San Diego, La Jolla, CA 92093 USA; 7 Department of Biological Sciences and BioDiscovery Institute, University of North Texas, Denton, TX 76203 USA

**Keywords:** [2Fe-2S], Iron-sulfur clusters, *Cisd(1*–*3)* encoded NEET proteins, NEET-fold, NEET-cluster lability

## Abstract

**Abstract:**

NEET proteins comprise a new class of [2Fe-2S] cluster proteins. In human, three genes encode for NEET proteins: *cisd1* encodes mitoNEET (mNT), *cisd2* encodes the Nutrient-deprivation autophagy factor-1 (NAF-1) and *cisd3* encodes MiNT (Miner2). These recently discovered proteins play key roles in many processes related to normal metabolism and disease. Indeed, NEET proteins are involved in iron, Fe-S, and reactive oxygen homeostasis in cells and play an important role in regulating apoptosis and autophagy. mNT and NAF-1 are homodimeric and reside on the outer mitochondrial membrane. NAF-1 also resides in the membranes of the ER associated mitochondrial membranes (MAM) and the ER. MiNT is a monomer with distinct asymmetry in the molecular surfaces surrounding the clusters. Unlike its paralogs mNT and NAF-1, it resides within the mitochondria. NAF-1 and mNT share similar backbone folds to the plant homodimeric NEET protein (At-NEET), while MiNT’s backbone fold resembles a bacterial MiNT protein. Despite the variation of amino acid composition among these proteins, all NEET proteins retained their unique CDGSH domain harboring their unique 3Cys:1His [2Fe-2S] cluster coordination through evolution. The coordinating exposed His was shown to convey the lability to the NEET proteins’ [2Fe-2S] clusters. In this minireview, we discuss the NEET fold and its structural elements. Special attention is given to the unique lability of the NEETs’ [2Fe-2S] cluster and the implication of the latter to the NEET proteins’ cellular and systemic function in health and disease.

**Graphical abstract:**

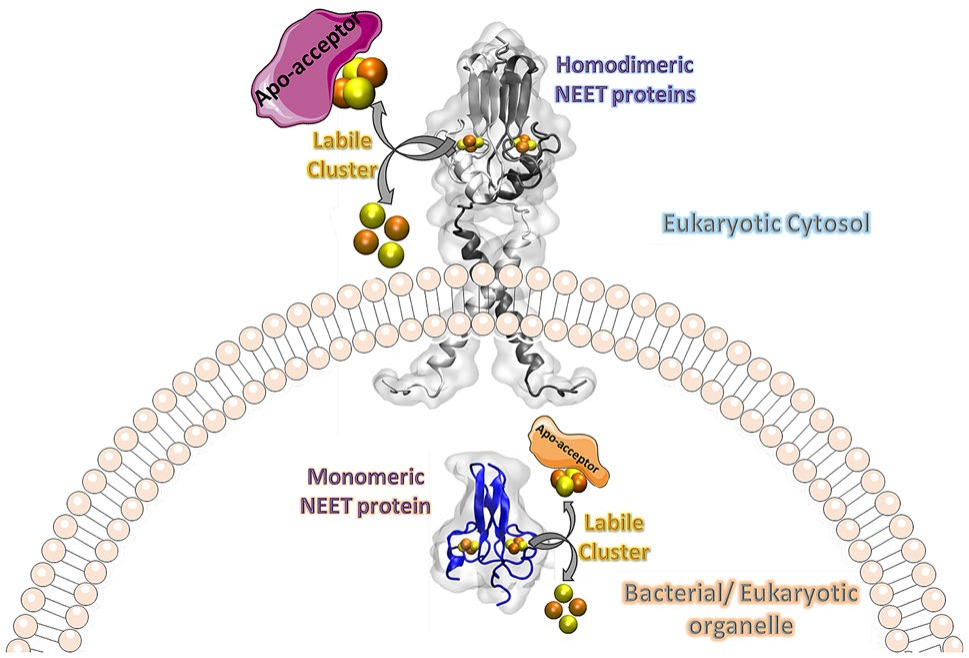

## Preface

Iron-sulfur (Fe-S) proteins play a crucial role in a wide array of biological processes including nitrogen fixation, photosynthesis and respiration [1–4]. These proteins are well characterized as electron transfer proteins [5]. However, in recent years, evidence for additional functions such as sensors of iron or oxygen [6, 7], enzymes [4], and gene expression regulation [8] were attributed to Fe-S proteins. In addition, in recent years, an increased number of human diseases were found to be associated with dysfunctions of the Fe-S cluster biogenesis pathway [8–11].

**Fig. 1:**
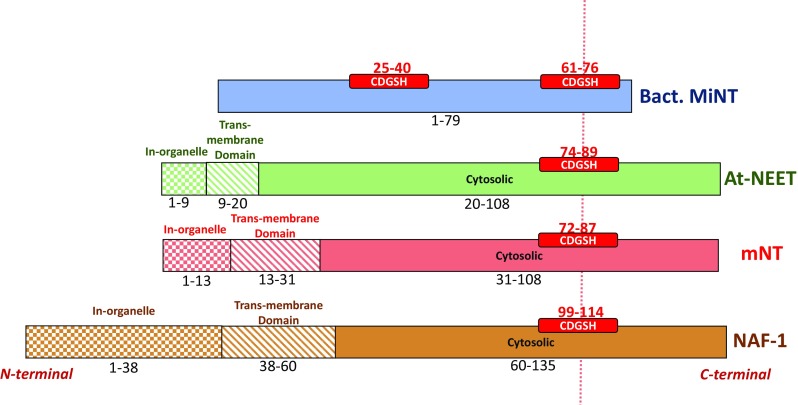
NEET proteins CDGSH organization. The location of the CDGSH domain(s) is shown in (red box) bacterial MiNT (blue), At-NEET (green), mitoNEET (red) and NAF-1 (brown). Different textures of the boxes were used to distinguish between different domains: in-organelle domain (checker texture), inter-membrane domain (diagonal lines pattern) and cytosolic domain (full color). The sequence interval is reported for each domain. The different regions specified here are based on the sequence of each protein

Recently, a new class of [2Fe-2S] proteins, the NEET protein family, was discovered [12–14]. The first member of this family to be identified was a mitochondrial protein mitoNEET (mNT) that binds the anti-type 2 diabetes drug pioglitazone. mNT is composed of 108 amino acids and is encoded by the *cisd1* gene [12]. The name of mNT and then of the entire NEET protein family is derived from the C-terminal sequence Asn-Glu-Glu-Thr (NEET) of mNT [12]. In a subsequent study [15] two additional members of the human NEET protein family were identified. These were the Nutrient-deprivation autophagy factor-1 (NAF-1; previously Miner1) which is composed of 135 amino acids and is encoded by the *cisd2* gene, and Mitochondrial inner NEET protein (MiNT; previously Miner2) which is composed of 127 amino acids and is encoded by the *cisd3* gene. NAF-1 was identified for its role in longevity [16] as well as for its association with several human diseases, neuronal development and the basic cellular processes of autophagy and apoptosis [13, 15, 17–24]. All three NEET proteins share a 39 amino acid sequence called the CDGSH domain (Fig. 1) [25]. The CDGSH domain contains a novel fingerprint motif, the 3Cys:1His cluster coordination motif of the [2Fe-2S] cluster domain which characterizes the NEET proteins [15, 26]. The human NEET proteins have all been shown to be associated with mitochondria; MiNT co-localizes with mitochondria while mNT and NAF-1 are located on the outer mitochondrial membrane (OMM) [15, 27]. The major parts of mNT and NAF-1 face the cytosol and a single transmembrane helix at their N-terminal region anchors each monomer of these homodimeric proteins to the OMM [25, 28, 29]. NAF-1 was also found on the ER- mitochondrial associated membranes (MAM) that connects the ER to the OMM, as well as to the ER [13, 27]. There is a high similarity between the different NEET proteins. In humans, mNT and NAF-1 share about 54% identical and 69% similar residues. In contrast, human MiNT shares about 50% identical and 63% similar residues with mNT, however, it has 38% identical and 50% similar residues to NAF-1 [30].

Phylogenetic analysis of NEET proteins indicates that the CDGSH domain has been conserved throughout the evolution of the NEET family. It is present in archaea and bacteria, mostly as monomeric proteins with two CDGSH domains [14, 31]. It has been suggested that the gene duplication that resulted in the eventual formation of mNT and NAF-1 in humans occurred around the time when vertebrates began to appear on Earth [31]. Furthermore, the closest CISD proteins to the ancient archetype of eukaryotic NEET proteins was proposed to be similar to NEET proteins of the slime mold *Dictyostelium discoideum* [31]. Since CISD proteins from snail, lancelet, hydra, lingual, sponge and sea anemone are more closely related to NAF-1, than to mNT, it has been suggested that NAF-1 evolved before vertebrates emerged and that mNT appeared via gene duplication after the radiation of vertebrates [31]. In addition, some organisms lost specific classes of CISD proteins, such as plants that do not contain MiNT-type NEET proteins. The CISD protein in plants, At-NEET (108 amino acids in length) (Fig. 1), resides both in chloroplast and mitochondria. At-NEET has a key role in plant development, senescence, reactive oxygen homeostasis, iron metabolism and homeostasis in different cells [30–32]. At-NEET encoded by the (At5g51720) gene shows 50 and 57% similarity to mNT and NAF-1, respectively, while its [2Fe-2S] binding domain has sequence identity to mNT and NAF-1 of about 75 and 88%, respectively [30, 32].

The present mini-review aims to emphasize the molecular components that contribute unique biophysical and biochemical properties to the NEET proteins. In particular, we describe two properties of the NEET proteins that affect their function, the unique ‘NEET fold’ and the structural elements of NEET proteins that determine the degree of liability of their [2Fe-2S] clusters. The implications of the latter in health and disease are also discussed.

## The unique ‘NEET-fold’ and structure

**Fig. 2:**
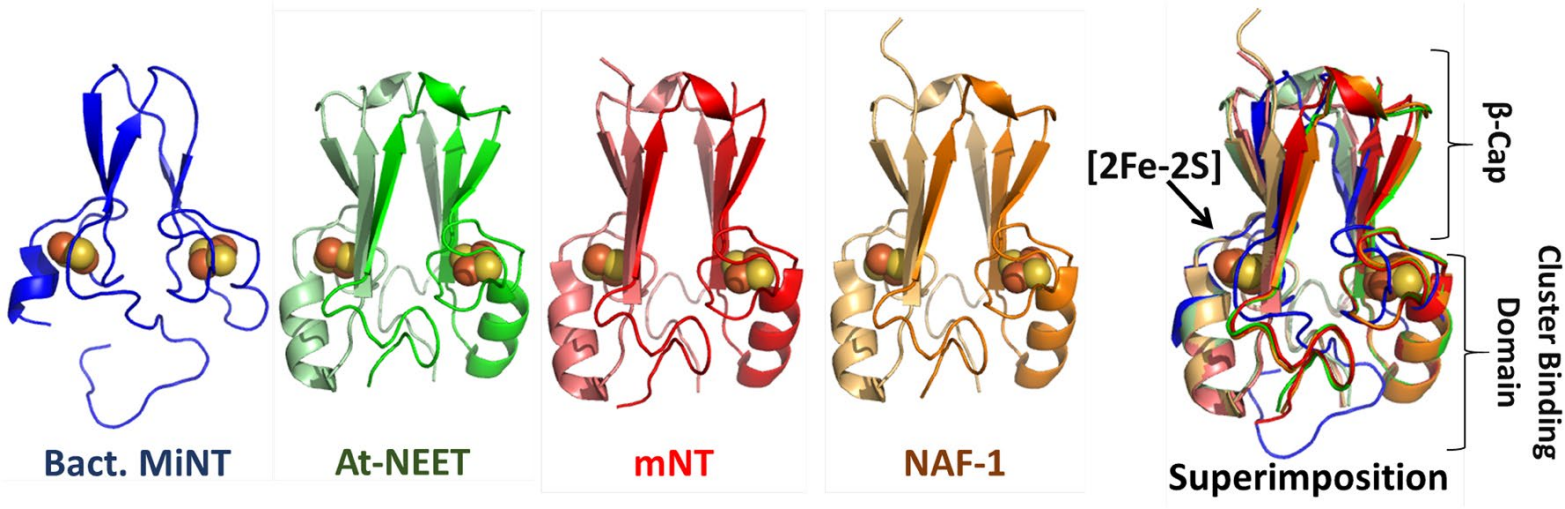
NEET proteins’ structures solved by X-ray crystallography. Structure of monomeric bacterial MiNT (blue colored; PDB-ID: 3tbn [14]), and dimeric (monomers A are reported with lighter colors) At-NEET (green, 3s2q [30]), mitoNEET (red, 2qh7 [15]) and NAF-1 (brown, 4oo7 [38]) proteins, and their superposition. The [2Fe-2S] cluster atoms are shown in orange-yellow spheres. The superposition of the four proteins shows the high structural similarity shared between the NEET proteins. The crystallized part of the homodimeric NEET proteins is limited to the cytosolic domain (fully colored part in Fig. 1) without the linkers to the membrane and the intra-organelle parts

The unique ‘NEET-fold’ [25] is highly conserved from bacteria through plants and humans NEET proteins [14, 30]. This fold and the NEET structures are unique compared to the 132,017 structures that have been deposited to-date, out of which 575 are known [2Fe-2S] proteins (http://www.rcsb.org) [25, 33] (Fig. 2).

To understand the physiological role of each NEET protein structure it is crucial to know the differences and similarities between each family member. Human mNT and NAF-1 as well as the plant At-NEET cytosolic structures have been well characterized [25, 28, 29, 34]. The MiNT structure we refer to is that of the *Magnetospirillum magneticum* bacterial homologue [14]. In this review, we used this structure for the comparison of the different NEET proteins, although the structure of human MiNT protein was published recently [35]. In contrast to the homodimeric proteins mNT and NAF-1, MiNT/Miner2 is a monomeric protein with two CDGSH domains (Figs. 1, 2) [14, 31, 35]. In mNT and NAF-1, each monomer contains a CDGSH domain, a trans-membrane helix and an in-organelle domain (Fig. 1). Since for the homodimeric NEET proteins only the soluble domains were crystalized, our structural comparison relates to the available structures (Fig. 2) [25, 28–30, 36–38].

### The ‘NEET fold’

All NEET proteins, including the bacterial monomeric MiNT that folds into a two-fold pseudo symmetric structure, are comprised of two main domains: a **β-cap domain** and **a cluster binding domain** (Fig. 2) [14, 25, 28, 29, 36, 37]. In the monomeric MiNT the β-cap domain comprises four β-strands. The β1 strand pairs with β4 forming a two stranded antiparallel sheet (see Table 1), while β2 pairs with β3 to form a second two stranded antiparallel sheet. These two β-sheets pack against each other and are linked by two loops between β1 and β2 (L2) and between β3 and β4 (L4). These two sheets comprise the β-cap domain. The cluster binding domains of MiNT are distinct and are characterized by three regions (L1, L3 and L5). The L3 loop is connected to the first [2Fe-2S] CDGSH coordination motif in the N-terminal (Cys25, Cys27, Cys36 and His40), and the L5 loop-helix is connected to the second [2Fe-2S] CDGSH motif in the C-terminal (Cys61, Cys63, Cys72 and His76) [14]. These two structural halves are connected by both hydrophobic and polar interactions. However, the backbone fold with its pseudo twofold symmetry is remarkably similar in all eukaryotic homodimeric NEET proteins (see Fig. 2 for the crystallized structures of the proteins; their sequences are contained in the fully colored part of Fig. 1) [14]. The backbone structures of the β-cap domains of the three eukaryotic NEET proteins (At-NEET, mNT and NAF-1) are highly similar [30]. In all three proteins, the β-cap domain primarily comprises three long β-strands per monomer (each containing 28 amino acids) [25, 29]. These β-strands are assembled in two symmetric β-sheets, which in each monomer are composed of two antiparallel strands from one monomer and a third parallel swapped-strand from the other monomer. The two β-sheets and the two linking loops on the top of the sheets (L2 in both monomers) form the β-cap sandwich domain (see Table 1 and see Fig. 2: The superposition comparison) [25, 29, 30]. The β-cap domain is held across from L2 by the cluster binding domain (Fig. 2). The cluster binding domain of each monomer contains a CDGSH domain (one per monomer), followed by an α-helix structure (see Table 1). The N-terminal of the soluble domain (L1) and the loop connecting the α-helix to the β-cap (L4) belong to the cluster binding domain. The structure of the N-terminus of NEET proteins, connected to the trans-membrane helices, is not yet known. However, the crystallographic structure of mNT showed that the cytoplasmic tethering domain could assume different orientations. This suggests high flexibility which may participate in protein–protein interaction [37], as well as affect the coupling between the folding and dimerization of NEET proteins [39, 40].

**Table 1:** The amino acids constituting the secondary structures elements and the protein domains of the NEET proteins

Protein	Cluster binding	*β*-Cap
Bact. MiNT	1–12, 25–48, 61–79	13–24, 49–60
At-NEET	44–59, 74–103	60–73, 104–108
mNT	42–58, 72–101	59–71, 102–108
NAF-1	68–84, 99–128	85–98, 129–135

	*β*_1_	*β*_2_	*β*_3_	*β*_4_	α_1_
Bact. MiNT	13–16	23–24	49–50	57–60	–
At-NEET	60–63	70–73	104–107	–	89–96
mNT	59–62	68–71	102–105	–	86–94
NAF-1	85–88	95–98	129–132	–	113–121

	Cys_1_	Cys_2_	Cys_3_	His
Bact. MiNT	25,61	27,63	36,72	40,67
At-NEET	74	76	85	89
mNT	72	74	83	87
NAF-1	99	101	110	114

The table details the amino acid indexes comprising the NEET cluster binding and β-cap domains (top panel); β-strands and α-helix of a single monomer (middle panel); and the coordinating residues of the [2Fe-2S] clusters (low panel). The Cys_i_ indexing follow the sequence of the conserved CDGSH domain. In MiNT the values of the two CDGSH domains of the protein are indicated. One should note that the amino acids relate to the structure of different NEET proteins solved by X-ray crystallography: At-NEET, 3s2q [30]; mitoNEET, 2qh7 [15]; NAF-1, 4oo7 [38]; bacterial MiNT, 3tbn [30]

Despite the very high level of similarity in the backbone structures, differences do exist between the AT-NEET, NAF-1 and mNT structures. For example, At-NEET and NAF-1 are slightly wider on the top of the loop connecting the intertwined strand to the other monomer in the β-cap domain, due to the presence of an extra amino acid in L2 that is not present in mNT (Asn69 in At-NEET and Thr94 in NAF-1) [29, 30]. In the crystalized structure of NAF-1, there is one free non-conserved Cys92 located to the upper part of the β-cap domain that was replaced with the isosteric Ser (C92S), due to instability and aggregation problems in the purification process [29].

### Differences in the repartition of the hydrophobic/charged residues in the homodimeric NEET structures

**Fig. 3:**
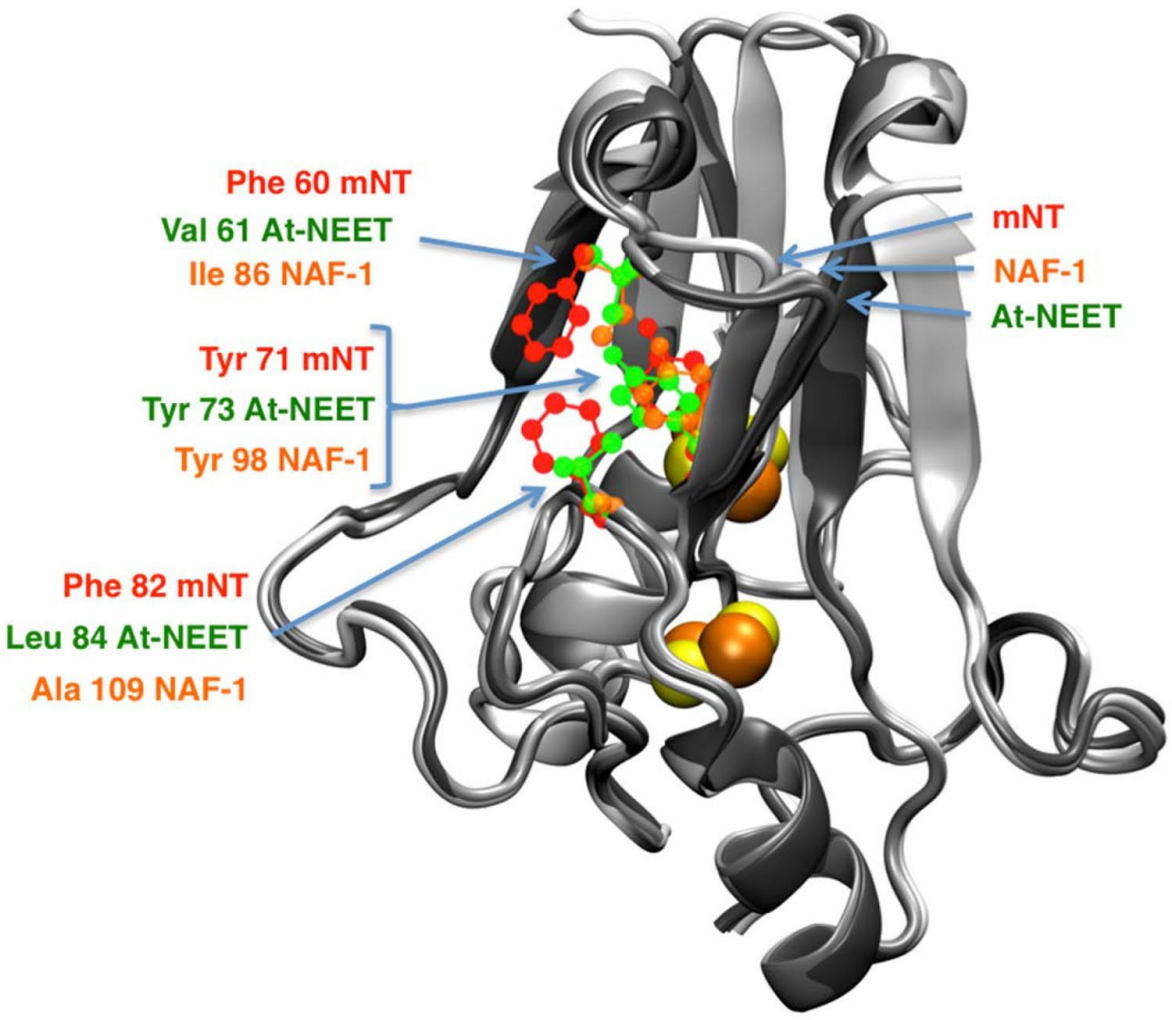
Central hydrophobic domains of the NEET proteins. The amino acids belonging to the hydrophobic central patch of mNT (red), NAF-1 (orange) and At-NEET (green) are shown in ball and stick representations over the structures of mNT [25], NAF-1 [38] and At-NEET [30] structures colored in grey shades from the brighter to the darker, respectively. The localization of the conserved Tyr is affected by the displacement of the surrounding amino acids, modulating, therefore, its position and orientation

The NEET proteins that are homodimers are stabilized by repartition of hydrophobic and charged residues [25, 30, 39]. Even though the distribution of the hydrophobic residues appears similar in all homodimeric NEET proteins, there are differences between the members [14, 29, 30]. On the surface of the mNT structure there is a convex hydrophobic ring that does not exist in At-NEET and NAF-1. This is composed of two Phe residues co-localized near the conserved Tyr. In contrast, At-NEET and NAF-1 have different hydrophobic residues co-localized to the Tyr which create a hydrophobic cleft in the same domain [30, 37] (see Fig. 3). The localization of Tyr is similar in all of the NEET proteins, when comparing root mean square displacement (RMSD) of the hydroxybenzyl group of Tyr, however, the level of similarity is highest between NAF-1 and At-NEET. There is a RMSD of 1.1 Å between NAF-1 (Tyr98) and At-NEET (Tyr73), whereas a RMSD of 1.5 Å exists between mNT (Tyr71) and either NAF-1 or At-NEET.

**Fig. 4:**
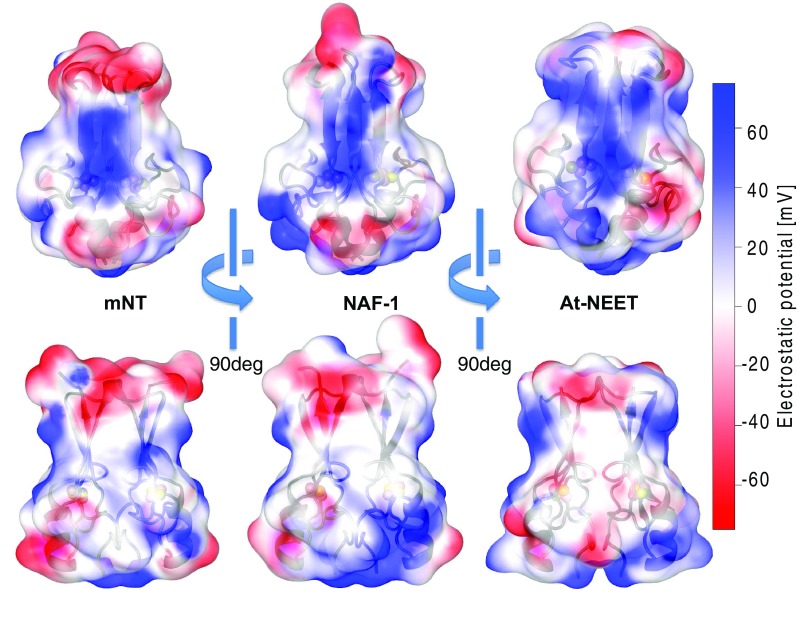
Electrostatic potential on the NEET protein’s surface. The electrostatic potential values (estimated using NEET proteins’ force files [85] and APBS electrostatic [103]) of mNT [25], NAF-1 [38] and At-NEET [30] are here reported over each protein surface. The side facing the plane of the β-sheet (top) and the side view (bottom) are here reported. The color code refers to the electrostatic potential values reported on the right

In general, the charged residues are distributed at the top of the β-cap domain and on the cluster-binding domain surface (see Fig. 4). This repartition of charged residues separated by the hydrophobic core (described above) leads to polarized/charged domains (depending on the family member) at the top and at the bottom of the two main domains of the proteins. In the folded part of the cytosolic domain of NEET proteins (residues 43–108 in mNT, 69–135 in NAF-1 and 44–110 in At-NEET), mNT is neutral, there is no net charge in electron units at pH 7.0, whereas NAF-1 and At-NEET both have a net positive charge (~ + 2 at pH 7.0) [41]. The electrostatics residues (marked onto the overlaid structures in Fig. 4) provide an insight to this change [14, 25, 29, 30].

Taken together, the differences described above for the structures and hydrophobic/electrostatic residues (Figs. 2, 3, 4), are associated with the variability in the homodimeric packaging, the amino acid composition and side chain orientation. For example, the mNT-Arg73 side chain, near the [2Fe-2S] cluster, forms an internal inter-monomer hydrogen bond with His58 side chain. The arginine is highly conserved across all the homodimeric NEET proteins (Arg100 inNAF-1 and Arg75 in At-NEET) [14, 30], while His 58 mNT is not conserved in NAF-1 and At-NEET. Intriguingly these Arg residues bind to the side chain of Asn84 and Asp59 of NAF-1 and At-NEET, respectively [29, 30]. This kind of difference in inter-monomer interactions can lead to differences in the stability of the dimeric structure among the NEET proteins. They may also affect different interactions of the NEET proteins and their partners and may also affect the lability/stability/redox potential of the [2Fe-2S] clusters [29].

### The cluster binding domain

The cluster binding domain, which is part of the CDGSH domain in all NEET proteins, harbors the [2Fe-2S] cluster. In general, the cluster binding domain is similar across species, with a higher sequence similarity compared to the other domains of the proteins and it is composed of the unique coordination of 3Cys:1His. The two [2Fe-2S] clusters interact by inter-cluster dipolar coupling [42, 43]. These clusters of the NEET proteins were shown to be redox-active. The redox properties can be tuned upon changes in the surrounding environment of the protein [26, 37, 44, 45]. Moreover, the [2Fe-2S] clusters may communicate via inter-dimer electron transfer, even when the clusters are at different oxidation states [43].

**Fig. 5:**
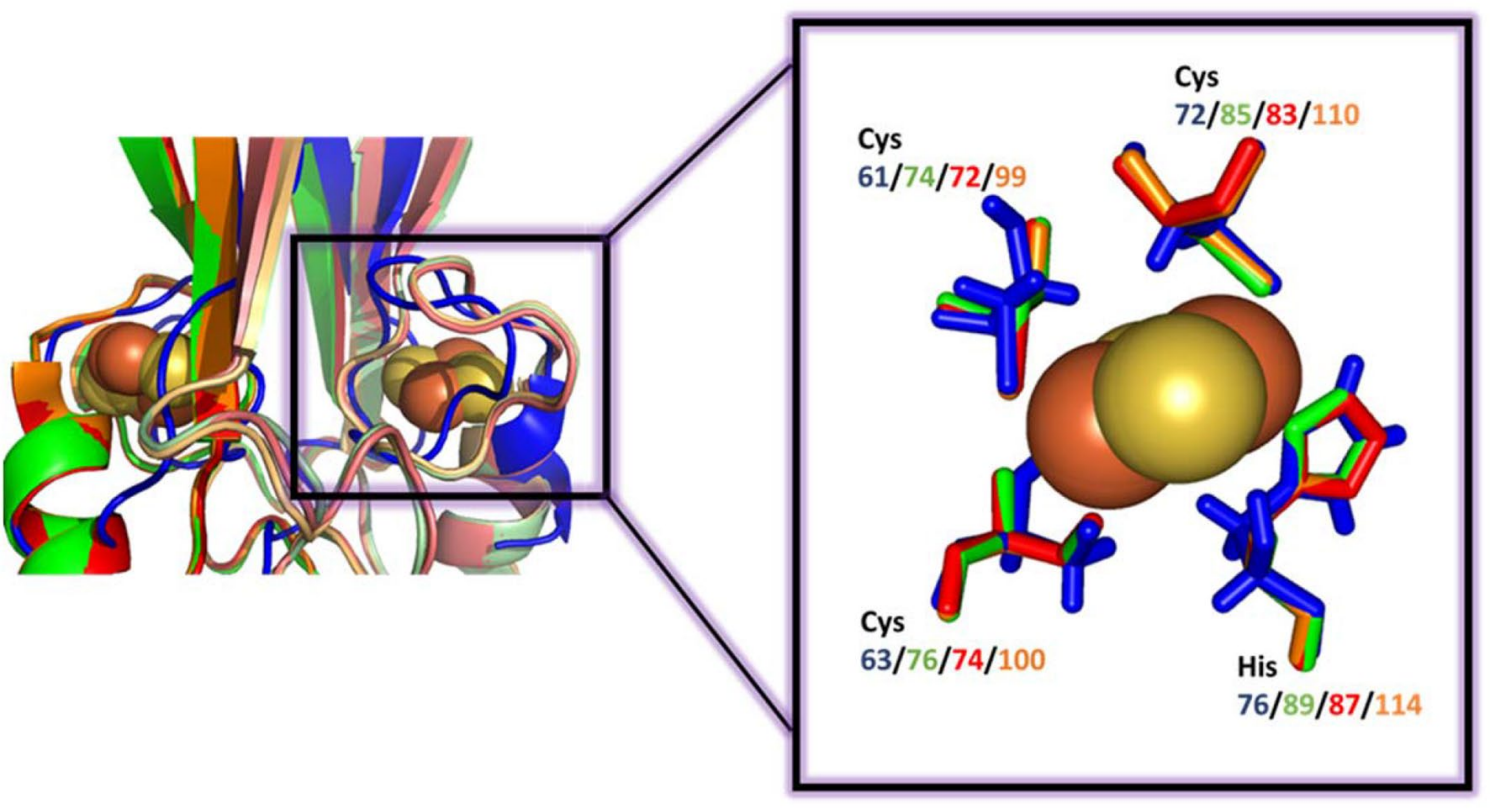
[2Fe-2S] cluster-binding domain of NEET proteins. Comparison of the superposition of the cluster-binding domain using the same color-code as in Fig. 2. The details of the superposition of the [2Fe-2S] 3Cys:1His pocket of each protein is shown within the box. In the left hand side figure the overlap between the proteins is not ideal for MiNT. Nevertheless, the similarity of the inner coordination sphere of ligands of the different NEET proteins is high

The [2Fe-2S] cluster coordinating His is located at the N-terminus of the α-helix within the cluster-binding domain. It is solvent accessible and it coordinates the outermost Fe of the [2Fe-2S] with one of the three Cys-ligands (see Fig. 5). The lability of the NEET [2Fe-2S] cluster is largely attributed to this residue (see the lability of the NEET cluster section, below). The last two Cys ligands coordinating the innermost iron of the [2Fe-2S] are buried inside the structure (see Fig. 5) [25, 29, 39].

## The labile [2Fe-2S] clusters of NEET proteins

Different biophysical and biochemical methodologies were used for the characterization of the [2Fe-2S] clusters of NEET proteins [26, 42–62]. These included UV–Vis absorption spectroscopy, mass spectroscopy (MS) [26], electron paramagnetic resonance (EPR) [42] and resonance Raman [45]. When the structure of the NEET proteins became available, it provided molecular–atomic explanations to the different biophysical measurements. For example, the MS of holo-mNT (mNT with the cofactor) vs. that of apo-mNT (mNT without the cofactor), obtained by lowering the pH, showed a molecular weight of 9230.6 (± 0.2) Da per hoho-mNT monomer and of 9056.9 (± 0.2) Da per apo-mNT monomer [26]. This indicated that the difference between the holo- and apo- forms of mNT is 173.7 (± 0.3) Da [26], which corresponds unambiguously to a [2Fe-2S] cluster. Indeed X-ray structures of the NEET proteins confirmed these findings. Moreover, biophysical studies made it possible to characterize the implications of the pH effects on the NEET proteins’ labile metal center [26].

The fingerprint absorption peak of the NEET proteins’ cluster in its oxidized form was found to be 458 nm; upon reduction this peak absorption is highly decreased [26, 39]. The ~ 90% decrease in the 458 nm absorption, under reducing conditions, can be fully recovered by exposing the NEET proteins to oxygen, proving that the [2Fe-2S] cluster of the NEET proteins is redox-active [7, 20, 26]. For a detailed description of the biophysical properties of the NEET cluster please refer to our previous review published by Tamir and his coworkers in BBA review—*Biochimica et Biophysica Acta (BBA)*-*Molecular Cell Research* [39].

We focus here on the molecular determinants of NEET proteins that contribute to the unique lability of their [2Fe-2S] clusters. The [2Fe-2S] cluster of all known NEET proteins are coordinated by 3Cys:1His. This feature distinguishes them from the highly abundant 4Cys coordinating structure of for example Ferredoxin, or the 2Cys:2His coordination of Rieske [39]. When first reported, this NEET protein’s coordination was unique among Fe-S proteins. In years to follow, e.g. in a D38A mutant of the iron-sulfur scaffolding protein IscU it was shown that the [2Fe-2S] cluster composed on the scaffold protein is also coordinated by 3Cys:1His [63]. Yet, crystal structure of the system indicated that while in NEET proteins the His coordinates the cluster via N_δ_, in the IscU protein the cluster is coordinated by the N_ε_ of the His [64].

When the structures of mNT and NAF-1 became available [25, 28, 29, 36, 37], the [2Fe-2S] clusters coordinating residues for mNT and NAF-1 were identified as Cys72/99, Cys74/101, Cys83/110 and His87/114, respectively [26, 29] (see Table 1, which also includes the plant At-NEET and bacterial-MiNT cluster coordinating residues). As Fig. 5 indicates, the superposition of the [2Fe-2S] cluster coordination sites of bacterial-MiNT through plant At-NEET and human mNT and NAF-1, form a nearly perfect overlay. The latter indicates that the cluster coordination site of NEET proteins was preserved through evolution from bacteria to human, which supports similar functional roles for the NEET proteins in all organisms. Importantly, differences in amino acid composition also create opportunities for selectivity in protein binding partners providing overlapping but not identical functions for multiple paralogs within a single organism (e.g., the cluster transferability between mNT or NAF-1 to anamorsin [55]).

The finding that NEET proteins have as a fourth coordinating cluster ligand, the His residue, which induces a pH-dependent-lability to their cluster, was proven as a unique feature of their [2Fe-2S] clusters. Indeed, the latter does not exist in Ferredoxins’ [2Fe-2S] (4Cys coordination) which have a high level of cluster stability under similar buffer conditions [26, 39, 45]. Moreover, when the coordinating His was replaced with a Cys, (H87C, H114C and H89C in mNT, NAF-1 and At-NEET, respectively), the [2Fe-2S] clusters of the NEET proteins were stabilized, similar to that of the Ferredoxin cluster. This stability is maintained in acidic pH [20, 26, 29, 30, 38]. In addition, the lability of the [2Fe-2S] cluster was shown to depend on the oxidation state of the cluster itself, and when the [2Fe-2S] cluster is in its reduced state in wild type His-containing NEET proteins, it is stable even at low pH [50]. This property may suggest that one of the functional roles of NEET proteins is to serve as a redox-sensing proteins [7]. Fe-S cluster containing proteins have the ability to play a role as sensors by losing their cluster, accommodating another type of cluster such as switching between [4Fe-4S] and [2Fe-2S], or receiving/transfering electrons, causing a change in the redox state of the cluster [65]. This sensing mechanism controls the activity of the Fe-S proteins in response to redox signals, through the changes of the redox state of their cluster [7]. Based on the cluster lability/stability studies we have suggested that NEET proteins are involved in ROS and Iron homeostasis [22, 30, 39, 66]. Recently, NEET proteins were also suggested to belong to Fe-S proteins that have a mechanism that when their [2Fe-2S] cluster are reduced the proteins are considered to be in a “dormant” state [7]; and when the cluster receives a signal that induces its oxidations, the NEET proteins are switched into an active state. The efficiency of this sensing mechanism may help cells to turn on their survival pathways quickly and recover from any stressful conditions [7].

As stated above, the pH-dependent stability of the NEET proteins’ [2Fe-2S] clusters, was associated with His protonation. Lowering the pH induced an accelerated loss of the clusters and its half-life was significantly decreased [26, 29]. To investigate the role of His in more details, His was replaced with Cys in mNT and NAF-1 proteins. Differences were observed between the mutants and their respective WT. Thus, the H114C-NAF-1 mutant structure shows the constant formation of a hydrogen bond between the Lys81 and Asn115 [38] while the H87C-mNT mutant structure showed two conformers having two distinct configurations for Lys55 and Cys87 [67].

This was also supported by the investigation of the residues surrounding His. In particular, the Lys that associates with mNT His87 (Lys55) plays an important role in conveying cluster lability, and the hydrogen bonding network helps to tune this stability, but not to affect the reduction potential [50]. The [2Fe-2S] cluster is also considered to be redox active with an Em value of 0 mV (± 10 mV) for mNT and NAF-1 at pH 7.5, the Em value is pH dependent and may decrease by approximately 50 mV per pH unit when pH is being increased from 7.5 to 10. This supports a mechanism whereby reduction is proton-coupled, and this often has a relevance to function [29]. As reduction is coupled to proton uptake, redox titration indicated that at a pH above the pKa of the oxidized state (pKox) and below the pKa of the reduced state (pKred), the reduction gives an uptake for a proton that is coupled to the His87 in mNT, which results in a pH-dependence vibrational interaction with the [2Fe-2S] center [47]. However, measurements of the protein redox potential and protein film voltammetry for the mNT [2Fe-2S] cluster were used to determine the pKa of the protein [44, 47, 50]. These models give pKa results of about 6.5 [44, 50] and 6.8 [47] for the oxidized [2Fe-2S] cluster. These models introduce empirical parameters that do not reveal the source of the proton donor and are not related to a specific amino acid. For this reason, further work is needed to investigate the protonation state of the coordinating His directly [68].

In addition to studies on the effects of pH on the cluster-lability, reduction of the coordinating His and pKa, the His to Cys mutations of the NEET cluster-coordination have also been found to affect the cluster redox potential (Em). Em can range from ~ 30 mV in wild type mNT/NAF-1 and about 0 mV in At-NEET, to ~ 10 times more negative values in mutants (> − 300 mV) such values are closer to the cluster Em of plant Ferredoxin (− 325 mV) and vertebrate Ferredoxin (− 235 to − 273 mV) [30, 39, 47, 69, 70].

Moreover, the variation between resonance Raman spectra of mNT protein and its Ferredoxin like mutant H87C which is found within peaks in the region of 250–300 cm^−1^ [45], support the hypothesis that the energy required for the cleavage of the Fe–N bond of a single His residue is modulated within the physiological pH range [45]. This may be considered as the first but not rate-limiting step prior to cluster loss, and in addition, this may be critical for in vivo functions of the NEET proteins [45]. This fact was further confirmed experimentally by the EPR study [42].

Several recent studies are focused on characterizing the electron transfer properties of NEET proteins and on the binding of the NEET proteins/[2Fe-2S] clusters to other small molecules. By mimicking the [2Fe-2S] harbor of NEET proteins in a model system, proton coupled electron transfer ability and the corresponding thermodynamic properties and function of the His ligand could be investigated.

Some studies focused on possible electron donors/acceptors for mNT [2Fe-2S] clusters in mitochondria such as flavin reductase which reduces flavin mononucleotides (FMN) to FMNH_2_ using NADH as electron donor. It was shown that mNT mediates the oxidation of NADH with concomitant reduction of oxygen [60, 61]. Interestingly, it was also shown that Fe-S clusters involved in Cys-coordination to protein are disrupted by nitric oxide (NO) [58, 71]. However, when the [2Fe-2S] clusters of MiNT are in a reduced state, MiNT can bind NO without disrupting the cluster. In addition, the other two human NEET proteins, mNT and NAF-1, fail to bind NO, but a single mutation, (D96V in mNT, or D123V in NAF-1) facilitates the binding of NO to the [2Fe-2S] cluster. This indicates that subtle changes to these proteins may switch their ability to bind NO, and thereby facilitate signaling in cells and modulation of mitochondrial function through NO signaling [58].

Despite the accumulation of valuable structural and molecular information on the ‘NEET fold’ as well as information on the structural and labile nature of the [2Fe-2S] clusters and how these affect NEET protein function, many issues remain to be solved. One such enigma is why mutations of amino acids that are at a large distance (more than 20 Å) from the [2Fe-2S] cluster, e.g. in the β-cap, affect the cluster properties (Em values, cluster transfer rates). Another concerns how cluster loss affects the structure of NEET protein. It was shown that cluster loss induces the unfolding of mNT [46, 52, 62]. However, nothing is known about the unfolding pathways of the NEET proteins. It is widely agreed that in the last two decades [72], molecular simulations have provided valuable insights into the structural determinants, the electronic structure and the spectroscopic properties of Fe-S proteins with [2Fe-2S] and [4Fe-4S] centers [72–78]. We here describe how theoretical simulation assisted in understanding some un-solved issues of the NEET proteins like the ones underlined above.

## Theoretical studies on NEET proteins

Computational studies have shed insights on the complex nature of the bonds between the Fe-S centers and the thiolated sulphurs of the Cys residues [72–84]. These studies used a partial or full application at the quantum mechanical level (QM). Full QM studies are usually performed on reduced domains of the protein or model systems representative of the region containing the cofactor. Since electronic processes can be affected by environmental effects, e.g. arising from the solvent and/or biomolecular frame, hybrid methods, combining QM and molecular mechanics (MM) allow for the electronic properties of the cofactor binding site to be characterised. In particular simulation studies have been extended to the bc1 protein complex [82, 83], that contains [2Fe-2S] clusters in which one of the two iron atoms is coordinated by two 2His residues. In addition, a study in which computational and experimental methods were coupled, was carried out on a model system mimicking the unique 3Cys:1His [2Fe-2S] cluster of NEET proteins. In this study, it was shown that concerted proton- and electron-transfer is involved in the process of reduction/oxidation of the [2Fe-2S] clusters [84].

Our team has recently applied established theoretical tools to study the peculiar coordination 3Cys:1His of the [2Fe-2S] cluster of the NEET proteins. The contribution of the different amino acids in the cluster binding region or in the distant β-cap domain to the clusters’ properties such as lability and reduction potential were studied. In addition, quantum mechanical calculations were applied to uncover key factors for the Fe–N bond’s reactivity leading to cluster liability [85].

**Fig. 6:**
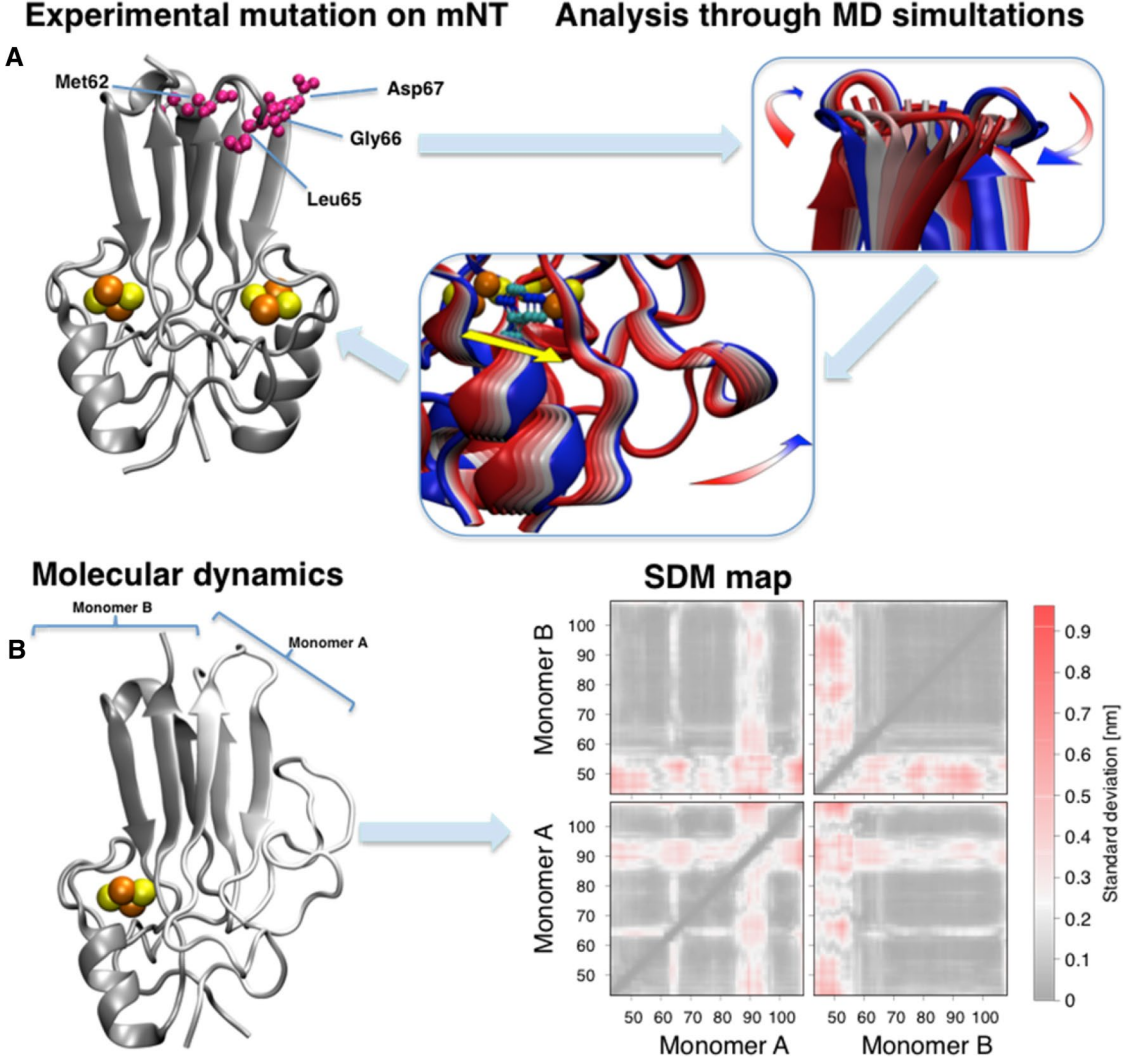
mNT modifications and theoretical analysis. (**a**, left) Crystal structure of mNT [25] highlighting the allosteric mutated residues at the top of the β-cap (violet). All crystal structures are available of the mutated proteins [51]. The β-cap mutations alter the coordinated motions of the domain (**a**, center panel), correlated with the flexibility of the cluster binding domain (right) and, in particular, with the coordinating histidine (yellow colored arrow) [51]. The colors of the cartoon structure in the central and right panels span from blue to red representing the movement along the principal vibrational mode of the protein. (**b**, left) Representative structure of the mNT in absence of one [2Fe-2S] cluster from monomer A obtained using replica exchange molecular dynamics [52]. (**b**, right) The effects of the cluster absence on standard deviation maps [52]. Here each pixel represents the standard deviation of the distance between each residue couple. The regions which are mostly affected by the cluster absence are the α-helix of the monomer losing the cluster and the L1 domain of the other [85]

Global structural information of mNT’s protein frame and the effects of chemical/physical properties of mNT on large time scales and spatial scales were uncovered [40, 51, 85, 86]. In particular computational studies on the mNT folding highlight the importance of the β-cap domain during the folding of the protein [40, 86]. An analysis of coupled regions on the folding landscape led us to predict where we could allosterically control the cluster properties from afar. This was followed by mutational and full structural analysis (see Fig. 6a) [51]. Mutations in amino acids of the β-cap domain affected the redox potential of the [2Fe-2S] cluster of mNT, less than the other mutations in amino acids that are proximal to the [2Fe-2S] cluster affect its redox potential [47, 51]. However, these mutations highly affected the mNT [2Fe-2S] cluster stability and cluster transfer rates [51]. Interestingly, cluster stability and cluster transfer rate were not correlated. X-ray structural analysis of the mutant proteins proved that the global fold of the protein remains unchanged. But, using energy landscape theory and all-atom structure based models, it was possible to understand that dynamic twisting of the β-cap domain result in scissoring of the distal cluster binding domain. The distal cluster binding domain altered the dynamic motions and transient distances between the coordinating His and the [2Fe-2S] cluster. Thus, while the global fold is maintained, changes in dynamic motions altered by mutations in sites that are 20 Å removed from the cluster, regulate cluster functional properties [51].

All atoms molecular dynamics on the NEET protein [2Fe-2S] cluster binding domain lead to two important suggestions. First, that the sensitivity to pH environmental variations [85] is mainly due to the differences between the amino acid that follows the coordinating His. This affects the localization of the conserved Lys (55 in mNT, 81 in NAF-1), which shields the His:Nε from the solven. Second, that conformational changes in mNT and NAF-1 are induced by a single (see Fig. 6b) or a double cluster release [85]. Upon the release of one cluster the α-helix of the monomer without the cluster is lost and, in addition, part of the structure of the other monomer is also affected. In case of loss of both the clusters, NEET proteins undergo a large structural rearrangement such as loss of both helices, along with the partial loss of the β-sheet structures.

In conclusion, the computational studies add valuable insight into questions raised by experimental results. Theoretical results also pose new questions for experimentalists. We believe that coupling experimental and theoretical investigations of NEET proteins will lead to detailed mechanistic information on the structure–function relationships, in particular with regard to the function of [2Fe-2S] cluster lability. Another major area in which computational tools are critical, is in clarifying the mode of binding of drugs to NEET proteins. The discovery of the NEET family was through mNT binding to pioglitazone [12], and more than a decade latter computational docking analysis resolved how pioglitazone stabilizes the [2Fe-2S] cluster [22]. The same holds true for other families of small molecules [87]. The drug design and binding studies are key for the pharmacological studies related to NEET proteins. Moreover, computational methodologies such as direct coupling analysis are also critical for defining NEET-partner proteins, e.g., NAF-1-BCL-2 [21] and for clarifying the cellular pathways that NEET proteins participates in.

## NEET protein involvement in diseases

NEET proteins are important in health and diseases. In healthy subjects, the *cisd2* gene, encoding NAF-1 protein, was shown to reside on chromosome 4. It is involved in longevity [16], and in mice several studies indicated that suppressed expression of *cisd2* led to shortened life spans [88]. In human pathologies mNT and NAF-1 were shown to be involved in diabetes and obesity [89, 90], neurodegeneration and cardiovascular abnormalities, and skeletal muscle maintenance [13, 29, 91], they were also implicated in autophagy, apoptosis [18, 21, 23, 92], aging [16, 93] and cancer [22, 23, 90, 94–97]. In addition, NEET proteins are implicated in the rare genetic disease Wolfram Syndrome 2 (WFS-2). In WFS-2 homozygous intragenic missense mutations lead to exon skipping introducing an early stop codon which results in the elimination of the NAF-1 protein from cells [13, 98–100]. Another genotype, leading to abnormal expression of NAF-1, was related to WFS-2 [101]. The phenotype of this syndrome is associated with hearing deficiencies, neurodegeneration, sever blindness, diabetes and a lower life expectancy [13, 99, 100].

The NEET proteins mNT and NAF-1 and recently human MiNT were shown to be involved in iron/Fe-S/ROS homeostasis in cells [23, 26, 30, 39, 49, 52, 55, 66]. These proteins, and in particular mNT and NAF-1, were found to function in the same pathways in mammalian cells [59]. By overexpressing mNT or NAF-1 in cells, activation of apoptosis and/or autophagy was prevented while cellular proliferation was supported by cellular resistance to oxidative stress [22, 96]. On the other hand, overexpression of the NAF-1 variant (H114C) did not promote cellular proliferation. In addition, such overexpression suppressed xenograft tumor growth [22]. Suppressing mNT or NAF-1 expression, results in over-accumulation of mitochondrial iron and ROS in mammalian cells, leading to the activation of autophagy and apoptosis [23, 39, 66]. This may be mediated through the interaction with other proteins; such as BCL-2 a key protein involved in autophagy/apoptosis regulation which is known to interact with NAF-1 [18, 21]. The interaction of NAF-1 with BCL-2 is thought to be controlled by the presence or absence of the [2Fe-2S] clusters of NAF-1 [18]. Since the presence or absence of the cluster in the protein may have a functional role in cells, it was important to evaluate the ability of the proteins to donate or accept clusters. This hypothesis was confirmed using different apo-accepters such as apo-Ferredoxin [20, 48]. By further investigating this ability it was critical to find physiological candidates for accepting the [2Fe-2S] cluster. The first one to be identified for both mNT and NAF-1 was Anamorsin, which is an electron transfer protein and is required for cytosolic Fe-S cluster assembly [55]. In addition, the mNT protein donates its clusters to cytosolic Aconitase [52, 102]. This was confirmed using the mutant forms of the protein H87C and H87S that replace the His with other amino acids and stabilize the cluster of the protein [102]. Another interaction between NEET proteins and the cytosolic Fe-S protein assembly machinery was through the redox switch mechanism of their clusters, through their ability to control the cluster transfer repair pathway, by transferring the NEET clusters to Anamorsin [57] and cytosolic Aconitase [52]. Most recently, mNT and NAF-1 which are known to maintain the levels of labile Fe and ROS were shown to be cooperating to control this homeostasis in mitochondria, and this result confirms the presence of a direct link between them. It may be that mNT transfers its cluster to NAF-1 and that this interaction regulates cellular proliferation and apoptosis/autophagy activation [59].

## Concluding remarks

This minireview focuses on the newly discovered [2Fe-2S] protein family, the NEET proteins, which are involved in numerous human pathologies and key cellular processes. We described in detail the unique fold and structural elements of these proteins and their uniquely labile [2Fe-2S] cluster that play a key role in their function. Although in the last decade a vast amount of information has been gathered about the NEET proteins, key questions related to the NEET proteins remain unsolved and await future studies. In particular, questions remain regarding structure–function, cluster lability, protein partner interactions and drugs binding. We strongly believe that coupling the experimental studies with computational simulations will pave the way toward answers to these questions and to the comprehensive characterization of this important NEET protein family.
